# Trait anxiety on effort allocation to monetary incentives: a behavioral and high-density EEG study

**DOI:** 10.1038/s41398-019-0508-4

**Published:** 2019-07-12

**Authors:** Cristina Berchio, João Rodrigues, Alina Strasser, Christoph M. Michel, Carmen Sandi

**Affiliations:** 1 Department of Basic Neurosciences, University of Geneva, Geneva, Switzerland; 2 Department of Mental Health and Psychiatry, Service of Psychiatric Specialties, Mood disorders unit University Hospitals of Geneva, Geneva, Switzerland; 3 Laboratory of Behavioral Genetics, Brain Mind Institute, School of Life Sciences, École Polytechnique Fédérale de Lausanne, EPFL, Lausanne, 1015 Switzerland; 4 Biomedical Imaging Center (CIBM), Lausanne, Switzerland

**Keywords:** Human behaviour, Neuroscience

## Abstract

Trait anxiety is an important phenotype in the prediction of stress-induced neuropsychiatric disorders. While the role of trait anxiety in mental effort and cognitive impairment is well documented, much less is known about its influence on motivated behaviors and physical effort. Here, we investigated trait anxiety-related differences in behavioral and neural responses in an effort-related monetary incentive delay task. Participants prompted with different incentive levels could exert handgrip responses to earn monetary rewards while a 256-channel electroencephalography (EEG) was recorded. Participants’ performance was linearly dependent on incentive level, with higher stakes prompting better accuracy and higher grip force. Importantly, we found a striking association between trait anxiety and incentive-related grip force; effort exertion was related to incentive level only in high-anxious individuals. In analyses of neural efficiency associated with effort preparation involving Contingent-negative variation (CNV), we found that the CNV amplitude was sensitive to monetary incentive levels. Source imaging analyses of CNV indicated increased activity in the anterior cingulate cortex (ACC) for the highest incentive level. Importantly, we found a significant interaction between trait anxiety and incentive level on CNV modulation at the interval ranging from −2610 to −2510 ms, with greater CNV responses to the lower monetary incentive sizes in high anxiety. Subsequent mediation analyses supported a mediation of the ACC activation on the association between trait anxiety and incentive-selective grip force. Our study reveals a role for ACC in trait anxiety-related differences on incentive processing, when rewards are dependent on effortful performance.

## Introduction

Individual differences in personality are crucial in determining human behaviors and are related to overall health and well-being^1^. In particular, trait anxiety is emerging as a highly relevant phenotype for the prediction of individuals at risk to develop stress-induced neuropsychiatric disorders, particularly anxiety disorders and depression^2,3^. Therefore, the identification of neurobiological mechanisms underlying variation in trait anxiety can advance our understanding of human behavior and disease vulnerability, and eventually illuminate the development of novel treatments.

Trait anxiety is a personality dimension related to the degree to which events, in general, are perceived as potentially threatening^4,5^. Trait anxiety has been shown to relate to several aspects of cognitive functioning^6–10^. Specifically, high-trait anxiety has been characterized by impaired attentional control under both threat^11^ and non-threat conditions^10^. However, despite these functional impairments, performance (i.e., effectiveness; typically measured as response accuracy) in demanding cognitive tasks is rarely impaired in a high-anxious individual [e.g., refs. ^12,13^]. Instead, high anxiety seems to impair processing efficiency (related to resource investment), as high-anxious individuals tend to take longer in completing cognitive tasks than low-anxious ones [for a review, see ref. ^14^]. Interestingly, a few studies have shown a differential neural engagement in high- and low-anxious individuals while solving cognitive tasks^15–17^. These findings support the view that in order to avoid negative consequences that may derive from poor task performance, anxious individuals make use of “compensatory strategies” (e.g., increases in effort or in use of resources) to boost performance^18^.

While evidence in support of this strategy has been well documented in the cognitive domain, much less is known about the influence of trait anxiety in other domains, such as motivated behavior or physical effort. Addressing this gap of knowledge seems highly relevant, given the increased risk of high-anxious individuals to develop depression^2,3^, a disorder frequently characterized by reduced motivation and anergia among its main manifestations^19,20^. The scarce evidence available from studies on sports performance has suggested that anxiety leads to increased self-reported effort^21,22^. However, the fact that trait anxiety is highly related to fatigue (in all its dimensions: general, psychological, and physical)^23–25^, raises the possibility that trait anxiety influences effortful motivated performance involving different incentive levels. Specifically, we hypothesize that effort allocation might differ between high- and low-anxious individuals, depending on reward expectancy.

A widely used paradigm to assess neural processing of reward is the monetary incentive delay (MID) task^26^. The MID task requires individuals to react to varying amounts of money and allows investigation of different stages of reward processing, including reward anticipation^27–29^. Monetary incentives can be gained or lost, cued or delivered, and prompted with different signals, such as the monetary amount possible to be won in a specific trial^30^. Not surprisingly, monetary incentives can predict motivated responses^31^ and energize behavioral outputs^32^. In some studies, an effort component is introduced in the MID task, frequently involving the squeezing of handgrip to earn a monetary reward^32–35^.

Functional magnetic resonance imaging (fMRI) and electroencephalography (EEG) studies have documented the recruitment of several brain regions—including the ventral striatum, insula, amygdala, and anterior cingulate cortex (ACC)—by anticipating rewards^28,29,36^, suggesting that a widespread network initiates motivational behaviors. EEG allows interrogating fine-grained temporal dynamics, including the dissection of sequential neural processes triggered by anticipation of different levels of monetary rewards^37,38^. One of the most widely investigated components related to expected values of future monetary rewards is the Contingent-negative variation (CNV)^39–41^, a slow preparatory wave elicited in the time interval between an alerting stimulus and an imperative cue^42^. The early part of the CNV has been related to the alerting properties of the warning stimulus, while the late part to cognitive anticipation and motor preparation^43^. Electrophysiological evidence indicates that the CNV activity may also reflect states of alertness^44^, and consistent with this notion, anxiety-related attentional biases seem to have a modulatory influence on its amplitude^44–46^. In particular, the amplitude of the early CNV was found to be greater in high-, but not low-, anxious subjects when vigilant toward negative, as compared with positive, affective stimulation^44^. Simultaneous fMRI and EEG studies have identified CNV generators in frontal regions, including the ACC^47^. Importantly, ACC has been linked to evaluating effort^35,48^, and identified as a critical signature of decision-making for choices involving motor costs^49^. In addition, ACC activation in some cognitive tasks has been shown to depend on trait anxiety^15^.

Here, we performed a study involving a modified MID task in which different incentive levels prompted participants to exert handgrip responses to earn monetary rewards while a 256-channel EEG was recorded. Our goal was to identify trait anxiety-related differences by comparing behavioral and neural responses between high- and low-trait anxious individuals. First, we asked whether trait anxiety relates to differences in behavioral strategies, and hypothesized differences in effort allocation prompted by three different incentive levels, but not in task accuracy. To this end, in addition to assessing accuracy, latency to respond and handgrip force [standardized for each participant with respect to his maximum voluntary contraction (MVC) force], we computed differences in participants’ results in each of these parameters between the higher and lower incentive (i.e., delta changes). Then, we interrogated the dynamics of brain activation following presentation of the different incentive levels. We expected that following the presentation of monetary incentives, participants would show augmented CNV responses to reward and source—the related brain activations, predicting the differential involvement of the ACC. We hypothesized that high levels of trait anxiety would be associated with the higher CNV amplitude (and ACC engagement) following presentation of the incentive/s in which group differences in effort allocation are observed. Finally, we developed a mathematical model accounting for the capacity of identified brain activations to mediate or moderate trait anxiety-related differences in behavioral strategies in the effortful MID task.

## Materials and methods

### Participants

The study involved 16 healthy male adult individuals. The sample size was determined by a power analysis with data from a previous pilot experiment (see Supplementary Materials). Table 1 contains participants’ phenotypic characteristics. For a complete description of participants and recruitment criteria, see Supplementary file.

**Table 1:** Descriptive statistics for participants’ Age, STAI-T, STAI-S, and MVC an MVC perceived mean and standard deviation separated by trait anxiety

	Low-trait anxiety (*N* = 8)		High-trait anxiety (*N* = 8)		*F*(1, 14)	*p*	η^2^
	*M*	SD	*M*	SD			
Age	23.75	3.84	23.00	2.82	0.20	0.664	0.014
STAI-T	31.50	3.70	48.75	6.07	47.14	<0.001	0.771
STAI-S	24.25	4.46	32.38	9.15	5.10	0.040	0.267
MVC	28.45	5.19	28.83	6.05	0.02	0.895	0.001
MVC perceived	80.0	16.90	81.25	14.58	0.03	0.876	0.002

Descriptive statistics are followed by the results of an ANOVA testing the effect of group on each variable

### Psychometric questionnaires

Participants were assessed for the state and trait anxiety, as well as state and trait fatigue. The trait-anxiety score was used to median-split the data into high- and low-trait-anxiety groups. For a complete description of psychometric questionnaires, see Supplementary file.

### Modified MID task

**Fig. 1:**
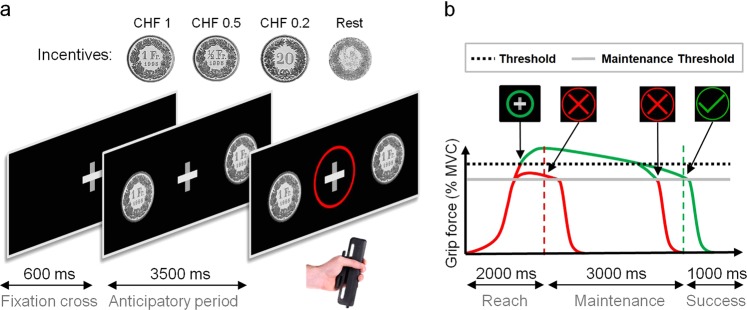
Incentivized and rest trials for the MID task. **a** Structure of the trial until the participant’s response. Each trial begins with a fixation cross followed by the appearance, alongside the fixation cross, of the incentive stimuli (CHF 1, 0.5, or 0.2) or a blurred coin face indicating a rest trial. The stimulus stays on-screen for an anticipatory period of 3.5 s, after which, a red circle appears around the fixation cross prompting the participant to act (except for rest trials) by squeezing the clench. The incentive remains on screen until the end of the trial and feedback about the participant’s response is given, using the color of the circle and fixation cross. **b** Possible outcomes for the participant’s response. Line colors represent the color of the enclosing circle and arrowheads point toward moments when feedback is given. Once the response is prompted, the circle remains red and participants have 2 s to reach the threshold of 40% MVC. If this is accomplished before 2 s, the circle turns green. Otherwise, a red cross replaces the fixation cross, indicating the failure of the trial. During the 3 s of the maintenance period, the circle remains green unless the exerted grip force drops below the maintenance threshold of 40% MVC—5 kgf, which results in the appearance of a red cross surrounded by a red circle signaling trial failure. If the maintenance period is completed above its threshold, the trial is successful and a green checkmark appears in place of the fixation cross during 1 s

Participants performed a modified version of the MID task^26^ (see Fig. 1). Monetary-incentivized trials started with a fixation cross (0.6 s), followed by an anticipatory period (3.5 s) indicating the potential gain: winning CHF 1, CHF 0.5, or CHF 0.2 (90 total incentivized trials). To earn these monetary incentives, participants were instructed to exert force on a hand dynamometer. The beginning of the force-exertion period was signaled by the appearance of a red circle around the fixation cross. If a certain threshold [i.e., 40% of the participant’s MVC force] was reached within 2 s, the red circle was replaced by a green circle. The green circle also indicated that participants had to maintain the contraction force level above a maintenance threshold (40% of MVC force −0.5 kg) for another 3 s. If participants did not reach the threshold in the initial 2 s or if the force level fell below the maintenance threshold during the 3 s endurance period, the trial was failed, visualized by a red cross occurring on the screen for 1 s. Trial success was indicated by a green tick (1 s) in the absence of the central fixation cross. To avoid preparatory biases during the fixation period, 30 resting trials, without monetary incentives, were also included. For a complete description of the task, see Supplementary file.

### Behavioral measures

Accuracy, grip force, and response latencies were measured, as well as variables representing the difference in behavior between the highest (1 CHF) and the lowest (0.2 CHF) incentive level were calculated for accuracy, grip force, and response latency and respectively termed ΔAccuracy, ΔGrip, and ΔLatency. For a complete description, see Supplementary file.

### EEG recordings and preprocessing

Scalp EEG was continuously recorded from 256 electrodes at 1000-Hz sampling rate (Electrical Geodesics Inc., Oregon). EEG pre-processing was performed using Cartool Software (https://sites.google.com/site/cartoolcommunity/)^50^. For a complete description, see Supplementary file.

### Hormonal responses

Participants’ saliva was collected to analyze salivary cortisol levels at both baseline and changes taking place during the experiment. For a complete description, see Supplementary file.

### Statistics

#### Behavior and cortisol

Repeated measures ANOVA (rm-ANOVA) was used to test for the effect of incentives on behavior. Mixed-design ANOVAs were applied to test for the interaction between incentives and trait anxiety on behavior. To analyze the interaction between incentive and state anxiety on behavior, repeated measures ANCOVAs (rm-ANCOVA), with state-anxiety scores as a covariate, were applied. For cortisol analyses, MVC or other variables not belonging to an incentive level, ANOVAs were used to test the main effect of the trait-anxiety group, and Spearman correlations to test the effect of state anxiety. A complete description and details of the post hoc tests and corrections for multiple comparisons applied can be consulted in the Supplementary file.

#### Electrical neuroimaging analyses

The CNV was analyzed using spatiotemporal methods^51–53^. To assess the strength of the responses to incentive levels at each time point, we used the global field power (GFP). The GFP measures the global strength of the electric field as the standard deviation of all potentials referred to the average reference^54–56^. It can be considered as a global measure of neuronal synchronization^57^. Differences of the GFP between the three incentive conditions were evaluated time point by time point using the RAGU software^58^ with an analysis of covariance (ANCOVA) with incentives (CHF 0.2, CHF 0.5 or CHF 1) as the main effect and anxiety as the covariate (trait-anxiety/state-anxiety scores). To prevent false positive rates, due to multiple comparisons across time, permutation tests were performed^59^. In addition, a post hoc analysis was applied to the average GFP of the identified time windows of significance (see ref. ^58^).

Source localization of the CNV was performed with Cartool Software^50^ using a distributed linear inverse solution ([LAURA^60^]. When covariant effects were significant, explorative post hoc analyses were performed (see the “Moderation and mediation” section) using the ROI identified by contrast analysis of incentives. For more details, see Supplementary file.

#### Moderation and mediation

To gain insight into whether and how brain activations affect the relationship between anxiety and behavior, moderation and mediation models were employed. The moderation analyses included a regression model containing (i) the behavior of interest as the response variable, (ii) the moderation between the brain activation of interest and trait anxiety, and (iii) other relevant confounding variables. One model was tested per ROI.

Similarly, we performed mediation analyses to test if any of the identified ROIs mediates the relation between trait anxiety and behavior. Elucidating this effect required the identification of a causal chain of trait anxiety predicting the CNV-related ROI activations, which in turn predict behavior. This analysis uses two regression models that were fitted separately: the mediator and the outcome model. In the mediator model, the mediator (the CNV activation at one ROI) is predicted by the treatment variable (trait-anxiety group) and a set of observed pretreatment confounders. In the outcome model, the outcome variable is predicted by the mediator, the treatment variable and the set of observed pretreatment confounders. One mediation was tested per ROI.

All *p*-values were corrected for multiple comparisons. For a complete description of these methods, see Supplementary file.

## Results

### Behavioral results

Table 1 displays the descriptive and inferential statistics for the two trait-anxiety groups (high and low), including information for age, trait and state anxiety, and MVC exerted on the handgrip to establish participant’s individual handgrip force thresholds. Both groups were equivalent in age while significantly different in trait-anxiety values. In addition, the high-trait-anxiety group showed a trend to display toward higher levels of state anxiety than the low-trait-anxiety group. However, state anxiety did not show significant effects on behavior (see Supplementary Results and Figure S1 in Supplementary Material for full details). Furthermore, the two groups did not differ for their respective MVC values, nor for their perceived MVC threshold. We also found that trait anxiety was positively correlated with trait physical and mental fatigue (see Table S1).

#### Accuracy

**Fig. 2:**
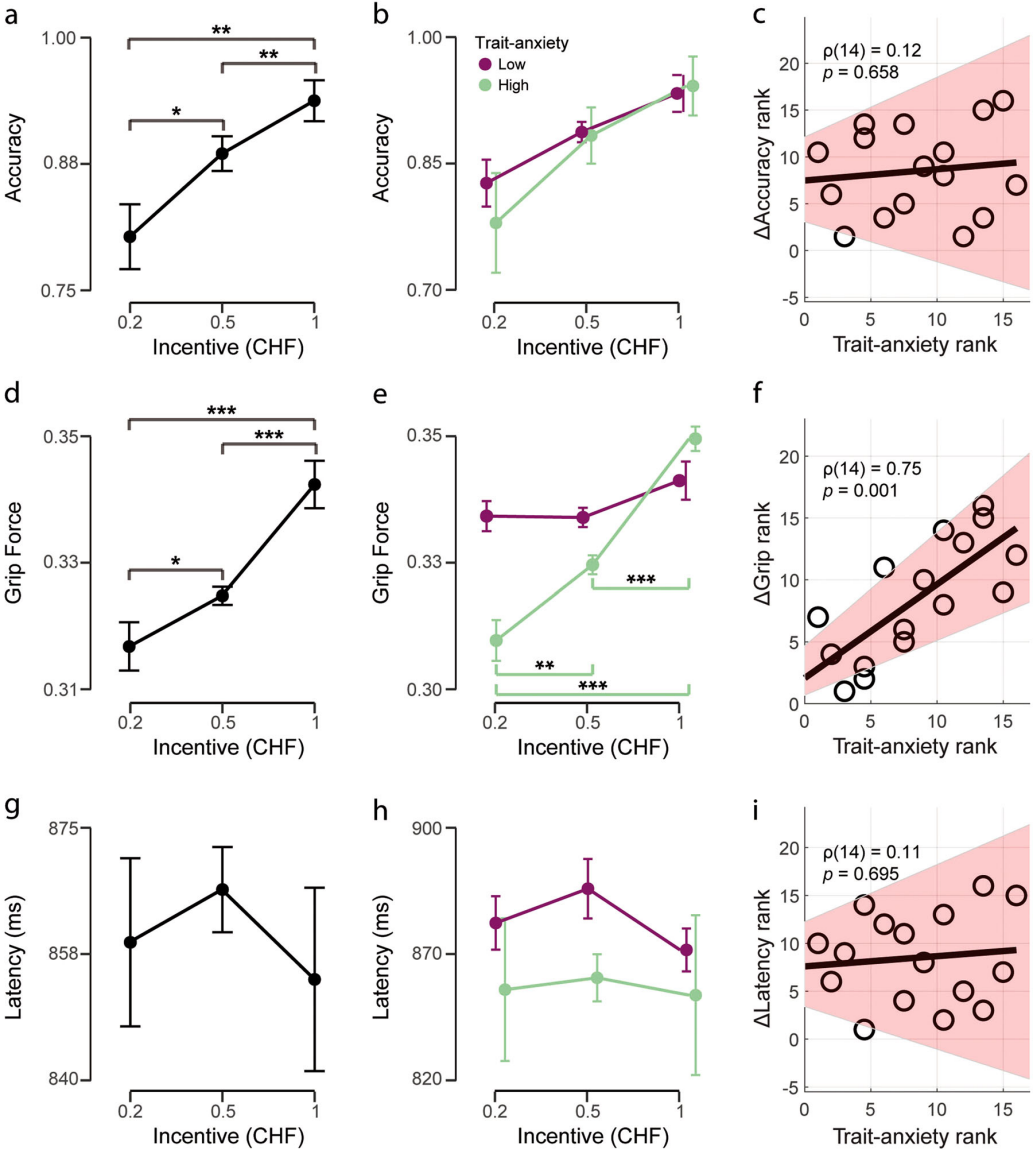
Behavioral effects of incentive level and trait anxiety. **a** Effect of incentive level on performance. High-incentive levels lead to increased performance. **b** No significant main effect of trait anxiety nor interaction effect between trait anxiety and incentive level were observed. **c** Spearman correlation between the variable ΔPerformance (performance CHF 1−performance CHF 0.2) and trait-anxiety scores. **d** Main effect of incentive level on grip force. **e** Interaction effect between trait anxiety and incentive level on grip force. High-trait-anxiety individuals respond to increasing incentives with increasing grip force. Post hoc statistics shown for the high-trait-anxiety group. **f** Spearman correlation between the variable ΔGrip (grip force CHF 1–grip force CHF 0.2) and trait-anxiety scores. High-trait anxiety leads to an increased difference in grip force between the CHF 0.2 and the CHF 1 incentives. **g** No main effect of incentive level on response latency. **h** Latency for high- and low-trait-anxiety groups for different incentive levels. No significant interaction or main effects were present. **i** Spearman correlation between the variable ΔLatency (latency CHF 1–latency CHF 0.2) and trait-anxiety scores. Significance levels: ***p < 0.001, **p < 0.01, *p < 0.05. Error bars represent standard errors of the mean (SEM). Shaded areas in Spearman correlation plots represent the bootstrapped 95% CI

As expected, accuracy in the MID task was affected by incentive level (see Fig. 2a; *F*_1.24,18.56_ = 7.95, *p* *=* 0.008, and *η*^2^ = 0.346). Specifically, accuracy for CHF 1 was significantly higher than for either CHF 0.5 or CHF 0.2, the latter being as well significantly inferior than accuracy for CHF 0.5 (all paired *t* tests *p* < 0.05, one tailed). Furthermore, no significant interaction between trait anxiety (low vs high groups) and incentive on accuracy was found (see Fig. 2b; *F*_1.24,17.40_ = 0.36, *p* *=* 0.605, and *η*^2^ = 0.016), nor a significant main effect of trait anxiety (*F*_1,14_ = 0.08, *p* *=* 0.786, and *η*^2^ = 0.005). Therefore, as expected from the absence of an interaction, there was no significant correlation between trait anxiety and ΔAccuracy (see Fig. 2c; *ρ*(14) = 0.12, *p* *=* 0.658).

#### Grip force

The total grip force applied in winning trials was affected by incentive level (see Fig. 2d; *F*_1.22,18.23_ = 16.78, *p* *<* 0.001, and *η*^2^ = 0.528). Post hoc paired *t*-tests revealed that the CHF 1 incentive leads to significantly higher grip force than CHF 0.5 [0.34 ± 0.02 vs 0.33 ± 0.02, respectively; one-tailed *t*(15) = 5.15, *p* *<* 0.001, and Cohen’s *d* = 1.288] and CHF 0.2 [0.34 ± 0.02 vs 0.32 ± 0.03, respectively; one-tailed *t*(15) = 4.22, *p* *<* 0.001, and Cohen’s *d* = 1.054] incentives and that in turn, the CHF 0.5 incentive leads to significantly higher grip force than the CHF 0.2 incentive [0.33 ± 0.02 vs 0.32 ± 0.03, respectively; one-tailed *t*(15) = 2.24, *p* *=* 0.020, and Cohen’s *d* = 0.559].

There was no main effect of trait anxiety in the total grip force (*F*_1,14_ = 0.61, *p* *=* 0.449, and *η*^2^ = 0.042). However, there was a significant interaction between trait anxiety and incentives (see Fig. 2e; *F*_1.46,20.38_ = 15.49, *p* *<* 0.001, and *η*^2^ = 0.248). Post hoc analyses indicated that a significant simple main effect of incentives on total grip force is present for the high-trait-anxiety group (*F*_2,14_ = 47.61, *p* *<* 0.001, and *η*^2^ = 0.248) but not for the low-trait-anxiety group (*F*_2,14_ = 1.92, *p* *=* 0.184, and *η*^2^ = 0.215). Specifically, for the high-trait-anxiety group, post hoc paired *t*-tests revealed that the CHF 1 incentive lead to significantly higher grip force than CHF 0.5 [0.35 ± 0.02 vs 0.32 ± 0.02, respectively; one-tailed *t*(7) = 19.46, *p* *<* 0.001, and Cohen’s *d* = 6.879] and CHF 0.2 [0.35 ± 0.02 vs 0.31 ± 0.03, respectively; one-tailed *t*(7) = 7.67, *p* *<* 0.001, and Cohen’s *d* = 2.710] incentives and that in turn, the CHF 0.5 incentive leads to significantly higher grip force than the CHF 0.2 incentive [0.32 ± 0.02 vs 0.31 ± 0.03, respectively; one-tailed *t*(7) = 3.15, *p* *=* 0.008, and Cohen’s *d* = 1.114]. For the incentive condition of CHF 0.2, the high-trait-anxiety group showed lower grip force than the low-trait-anxiety group [0.31 ± 0.03 vs 0.33 ± 0.02, respectively; one-tailed *t*(14) = 1.83 *p* *=* 0.044, and Cohen’s *d* = 0.914].

We also found a significant positive association between trait-anxiety scores and ΔGrip [see Fig. 2f; *ρ*(14) = 0.75, *p* *=* 0.001]. This effect is specific to trait anxiety since no significant effects were found for state anxiety (see Supplementary Materials).

Importantly, total grip force was not associated with accuracy. No significant correlations were found between accuracy and total grip force for each incentive level, nor between ΔGrip and ΔAccuracy (all *p*s > 0.636 for the four correlations; see Table S1).

#### Response latency

There was no significant main effect of incentive on response latencies (see Fig. 2g; *F*_1.34,20.16_ = 0.35, *p* = 0.622, and *η*^2^ = 0.023). No interaction between incentives and trait anxiety (see Fig. 2h; *F*_1.34,18.68_ = 0.10, *p* = 0.823, and *η*^2^ = 0.007) was found, nor significant main effects of trait anxiety (*F*_1,14_ = 0.16, *p* *=* 0.699, and η^2^ = 0.011). Trait anxiety was not significantly associated with ΔLatency (see Fig. 2i; *ρ*(14) = 0.11, *p* = 0.695).

No significant correlation was found between ΔLatency and ΔAccuracy or ΔLatency and ΔGrip (all *p*s > 0.966; see Table S1).

### Electrical neuroimaging

#### Analysis of CNV strength

**Fig. 3:**
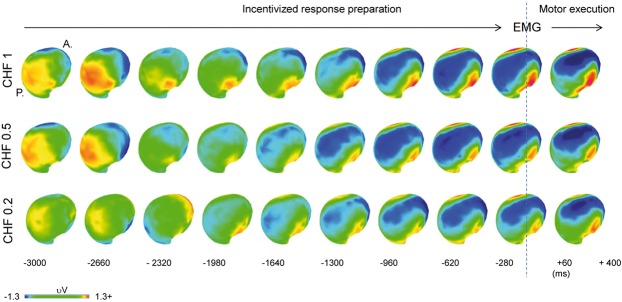
Topographic CNV maps during incentivized response preparation. Negative values are represented by blue/purple colors; positive values by yellows/reds. For all incentives, CNV development shows a central negative maximum coincident with the EMG onset. During response preparation, inspection of maps highlights differences between incentives, particularly pronounced at early stages and for CHF 0.2

**Fig. 4:**
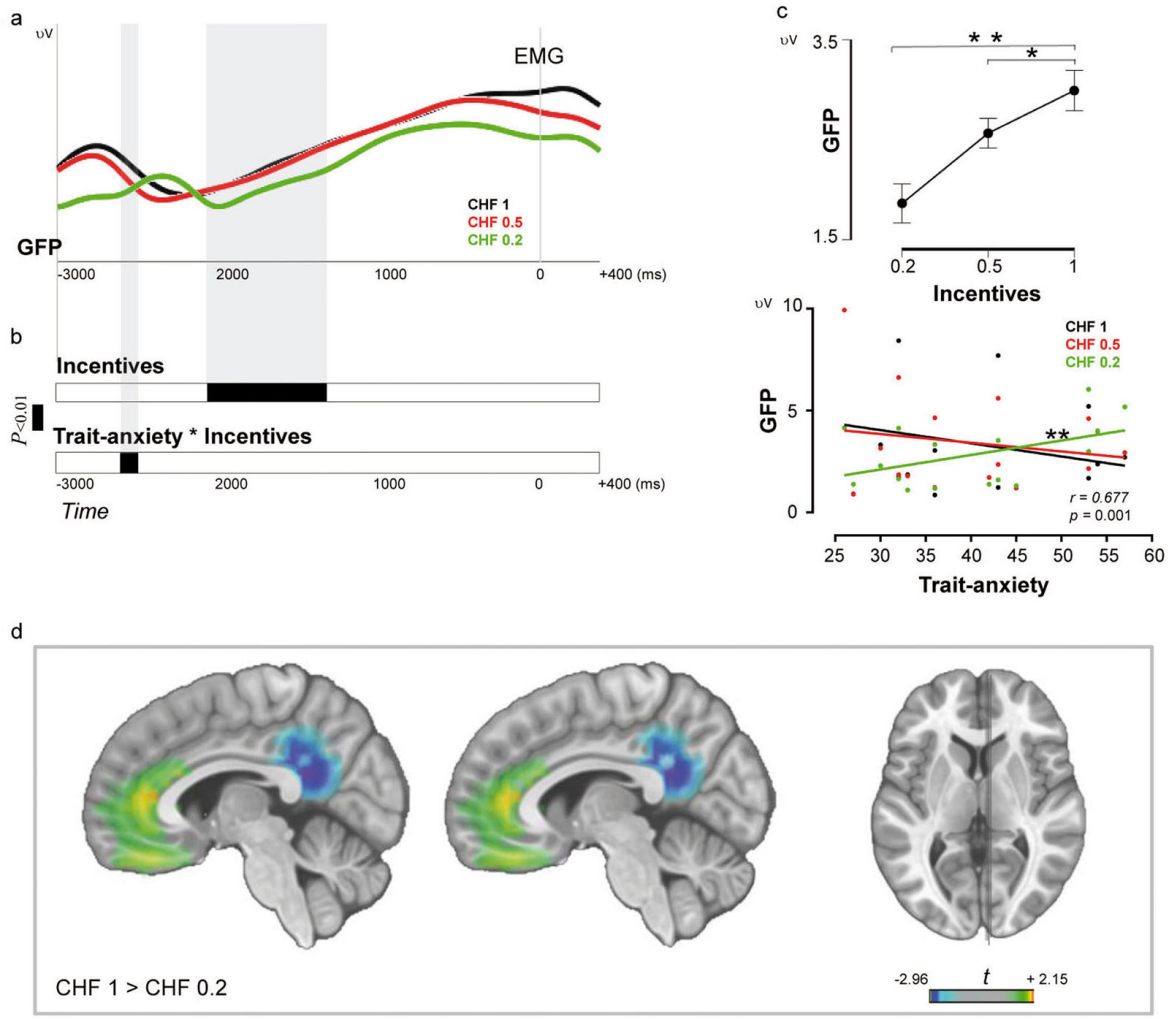
CNV modulations. **a** Incentivized response preparation evoked by three monetary incentives. The strength of the responses to monetary incentives is shown by the global field power (GFP). **b** Waveform analyses on the GFP. The results of the ANCOVA indicated a main effect of incentive level and a significant covariance effect of trait anxiety. Black solid lines indicate the presence of significant effects, and temporal modulations are highlighted by light gray bars (*p* *<* 0.01). **c** A main effect of incentive level indicated that the highest incentives (i.e., CHF 1 and CHF 0.5) elicited larger GFP amplitudes than CHF 0.2 (**p < 0.01, *p < 0.05), and that the strength of the cortical response was positively correlated with trait anxiety for the CHF 0.2 incentive (**p < 0.01). Error bars represent SEM. **d** Electrical neuroimaging analyses: main effect of incentive level (*p* < 0.05). Red–yellow colors indicate that brain regions are activated strongly during response preparation for CHF 1, blue–purples during response preparation for CHF 0.2

Figure 3 shows the scalp topographic maps, across all participants, in response to incentives anticipation. As described previously^43^, CNV topographies appear as a central negativity and temporal positivity. This topography begins to develop at around −2320 ms before movement onset for CHF 1 and progressively later for CHF 0.5 and CHF 0.2, respectively. Differences in CNV strength (GFP) between incentives were evaluated for each time point using ANCOVA with anxiety measures (trait/state anxiety) as a covariate (Fig. 4a, b). The ANCOVA revealed a significant interaction between trait anxiety and incentive level at the interval ranging from −2610 to −2510 ms (*p* *<* 0.01). Significant correlations were identified by bootstrap statistics (*R* model: 0.677; *p* model = 0.001). Specifically, the strength of the CNV in this time window was positively correlated with trait anxiety for the CHF 0.2 incentive (*r* = + 0.688, *p* = 0.004; all other *p*_s_ > 0.6). In addition, ANCOVA revealed a main effect of incentive level ranging from −2115 to −1345 ms; *p* *<* 0.01. A *post hoc* *t*-tests of the GFP across the whole time window indicated significantly higher CNV amplitudes for CHF 1 (*M*: 2.989; *SD*: 2.121) as compared with CHF 0.2 (*M*: 1.861; *SD*: 1.162) (*p* *=* 0.002), and for CHF 0.5 (*M*: 2.562; *SD*: 1.483) as compared with CHF 0.2 (*p* *=* 0.010). No differences were found between CHF 1 and CHF 0.5 (*p* *=* 0.086) (Fig. 4c).

#### Analysis of CNV source activity

To localize the brain network associated with response preparation, we compared the conditions at high contrast (CHF 1 vs CHF 0.2) in the time window corresponding to the main effect of incentives (−2115 to −1345; see Fig. 4a). Source strength was averaged across this time window and across solution points within each of the 80 ROIs. Randomization tests were then performed for each ROI with a significance level set at *p* < 0.05. Differences between conditions were found in the ACC and in the PCC (Fig. 4d). Unpaired *t*-tests indicated that a high level of incentive (CHF 1) induced increased right ACC activity (*t* = +2.15, *p* = 0.0481), while low incentive (CHF 0.2) induced augmented activity of the PCC bilaterally (right hemisphere: *t* = −3.20, *p* = 0.005; left hemisphere: *t* = −2.969, *p* = 0.009) (see Fig. 4). Based on the results of the main incentive comparison, and also by strong evidence that these areas are key regulatory nodes of reward, effort, and action encoding^49,61^, the ACC and the PCC regions were then used for subsequent analyses.

### Hormonal responses

No significant effects of trait anxiety were found for cortisol AUCg (low-trait anxiety: 1.00 ± 0.33 vs high-trait anxiety: 0.78 ± 0.19; *F*_1,14_ = 2.80, *p* = 0.117, and *η*^2^ = 0.167) nor for cortisol AUCi (low-trait anxiety: −0.47 ± 0.58 vs high-trait anxiety: −0.25 ± 0.36; *F*_1,14_ = 0.88, *p* = 0.364, and *η*^2^ = 0.059). Cortisol variables were not directly associated with behavior (accuracy or grip force) or brain activations.

### Brain moderation and mediation effects on behavior

To assess whether incentive-driven brain activations relate to variation in delta grip force (ΔGrip) associated with trait anxiety, we tested different models representing both mediation and moderation effects (see “Methods”). The three identified ROIs associated with the CNV (left and right PCC for CHF 0.2 and right ACC for CHF 1) were tested separately as possible moderators and mediators between trait anxiety and ΔGrip. Brain activations selected for the model corresponded to the time interval, where the interaction between trait anxiety and incentives significantly predicted CNV responses (i.e., from −2610 to −2510 ms). Since reporting high behavioral self-confidence might result in the higher grip force during the 0.2 CHF responses (see Table S1) we added this variable as a confounder for the regression models.

In three different moderation models, no moderation effects were found between trait anxiety-related activations and ΔGrip (all *p*_s_ > 0.649).

Conversely, in the three mediation tests, the right ACC was identified as the only significant mediator (ACME values were compared for the three models) in the relation between trait anxiety and ΔGrip (ACME; *p* = 0.44, all other *p*_s_ = 1). Both the mediator and output regression models passed linear model assumptions, skewness, kurtosis, and heteroscedasticity tests. The mediator regression model had an adjusted *R*^2^ of 0.3 and within this model, the regressor for the trait-anxiety group had a large effect predicting activity in the ACC [*β* = 0.09, SE = 0.04, *t* = 2.68, and *p* = 0.019] while confidence was not significant (*p* = 0.150). In the output regression model (adjusted *R*^2^ of 0.76) the regressors for the trait-anxiety group [*β* = 0.02, SE = 0.01, *t* = 3.05, and *p* = 0.001], ACC [*β* = 0.11, SE = 0.04, *t* = 2.65, and *p* = 0.021] and confidence in task performance [*β* = −0.01, SE < 0.01, *t* = −3.83, and *p* = 0.002] predicted ΔGrip significantly.

**Fig. 5:**
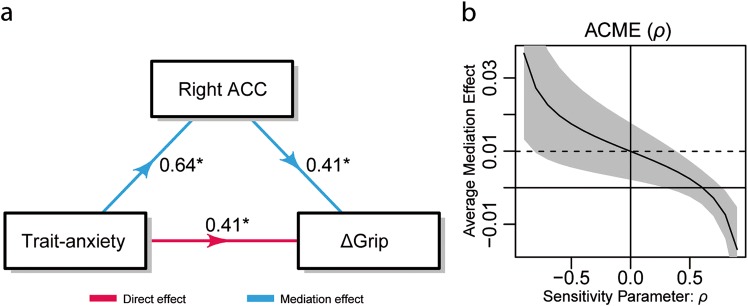
Complementary mediation effect of right ACC activations for CHF 1 between trait anxiety and ΔGrip. **a** Path diagram for the mediation effect with standardized regression coefficients. **b**) Sensitivity analysis. The solid line represents the estimated ACME for the attitude mediator for differing values of the sensitivity parameter *ρ* and the gray region represents the 95% confidence interval. The horizontal dashed line is drawn at the point estimate of the reported mediation effect, under the assumption of sequential ignorability. Significance levels: *p < 0.05

Both the ADE and the ACME were positive and significant (ADE *=* 0.02, *p* *=* 0.025; ACME = 0.01, *p* = 0.044), and the mediation effect accounted for 32.3% (*p* *=* 0.017) of the significant total effect. Thus, there was a complementary mediation effect of the right ACC between trait anxiety and ΔGrip (Fig. 5a). Figure 5b presents the results for the sensitivity analysis based on the residual correlation *ρ*. The estimated ACME is plotted against the changing values of ρ, which assumes the value of 0.61 when ACME is zero (95% CI [0.30, 0.77]). This indicates that the assumption of sequential ignorability for the estimated ACME would be maintained unless *ρ* is larger than 0.61, implying that the moderation effect is reasonably robust to the violation of this assumption.

## Discussion

Here, we applied an effortful handgrip MID task combined with high-density EEG to investigate whether trait anxiety influences behavioral responses and to elucidate the associated brain activity following incentive cues of different size. In agreement with our prediction, we found key differences in effort (i.e., exerted handgrip force) allocation due to trait anxiety across task incentives. Thus, in addition to revealing lower grip force exerted by high- compared with low-trait-anxious individuals for small incentives, we found a striking association between trait anxiety and ΔGrip. Specifically, the high-anxiety group displayed differences in effort allocation across different incentives (i.e., lower force for lower incentives), while low-anxiety individuals showed similarly “energized” behavior across incentive levels.

Rewards motivate operant behavior, and incentives that signal the possibility to obtain different reward levels can energize behavior to different degrees^62^. Indeed, individuals tend to exert more effort to obtain greater rewards^32^. In our modified MID task, effort requirements were maintained constant across different incentives; i.e., the same grip force was required to earn the different rewards. However, it is important to note that while the minimum force exertion was set at 40% MVC, there was neither upper limit nor feedback regarding the maximum handgrip force exerted by participants in each given trial. As expected, participants’ performance in our study was linearly dependent on incentive level, with higher stakes leading to higher accuracy and grip force. In addition, as hypothesized, we did not observe trait anxiety-related differences in accuracy, which aligns with evidence for a lack of accuracy decrements in high anxiety in the cognitive domain [see the “Introduction” section^12,13^].

The fact that the ΔGrip gradient for different incentive sizes was only present in high- but not low- trait anxiety reveals major differences in the behavioral strategy these two groups solved this effortful MID task. Since we did not ask participants about whether they applied a particular strategy to solve this task, it is not possible to disentangle the contribution of conscious versus unconscious decision-making processes impinging on the “economy” of effort allocation. Indeed, studying the contribution of conscious versus unconscious aspects of effort allocation would furthermore have required a different experimental setup (see for instance^32^). Instead, not mutually exclusive explanations might account for our findings. First, our task did not vary grip force requirements for the different reward cues, and thus our results might be explained by group differences in cost-benefit assessment. Specifically, processing of the monetary incentive size, and consequently its behavioral impact, seems to be strongly modulated by anxiety. In low anxiety participants grip force was similar for the different incentive sizes, while in the high-anxiety participants, grip force reflected a higher sensitivity to varying incentive levels and their consequent energizing nature. Accordingly, high-anxious participants would be ascribing a lower value to the low incentive (CHF 0.2) than low-anxious ones. This finding is in line with the phenomena of response effort discounting on subjective values of potential rewards^63^. Although we cannot discard a potential contribution of differences in effort perception, high- and low-trait anxiety participants did not differ in their perception of the grip force threshold used in the task. However, previous studies, involving subjective assessments, have shown that high-anxious individuals tend to report more subjective effort investment when performing physical tasks than low-anxious ones^22,64^, suggesting that anxiety impacts on effort weighting, with the neurobiological underpinning not having been reported previously. In fact, an increase of effort “cost” in high-anxious subjects would be in line with the reported capacity of a chronic treatment with the antidepressant/anxiolytic escitalopram to increase handgrip force production in participants working toward monetary benefits^65^.

A second possible explanation of the observed group differences is the idea that high- and low-anxious individuals differ in their respective “energetic resources” in task associated physiological systems, and differentially activate functional components necessary for behavioral responses in physical effort tasks, such as our modified MID task. Accordingly, high-anxious individuals might have developed a performance strategy in order to maximize monetary earnings while saving energy expenditure for the lower stakes. In this context, it is relevant to highlight the recently reported negative association between trait anxiety and physical fatigue, with taurine metabolite concentrations in the nucleus accumbens (NAc)^24^. The NAc is a hub for the regulation of motivated behaviors^66^ and effort exertion^19,67^, and taurine, an abundant amino acid, plays key regulatory functions (e.g., neuromodulation, osmoregulation, membrane stabilization, and antioxidant action) in the brain^68^. Furthermore, evidence from rodent studies highlights impaired energy metabolism and mitochondrial function in the NAc of high-anxious animals^69–72^, supporting the view of a potential “limited resources” principle underlying motivational decisions and effort exertion in high-anxious individuals. Interestingly, in connection with the discussion below on ACC, there are reciprocal projections between the NAc and the ACC^73^. Thus, although data on energy metabolism in the ACC are still missing, variation in metabolic resources in the NAc may affect, through these connections, the degree of ACC engagement and functioning.

The view that a “limited resources” explanation might underlie the reduced grip effort exerted by high-anxious participants for low incentives is in line with a potential involvement of increased fatigue in these individuals. In agreement with the literature^23–25^, we report here an association between trait anxiety and traits of physical and mental fatigue. Fatigue is a feeling of exhaustion that typically deters performance by affecting processes of cost-benefit analysis of effort exertion [for a review see ref. ^74^]. Interestingly, the ACC is one of the key brain regions identified by neuroimaging studies underlying behavioral changes due to fatigue^74^. The aforementioned, together with the fact that we observed increased grip force in high anxiety for greater incentives, highlights that incentives can counteract fatigue in both physical and cognitive domains^74,75^. How NAc taurine levels might be implicated in cognitive cost–benefit analysis might be an important future research question to be addressed, since we have recently reported a negative association between NAc taurine metabolite concentrations and physical fatigue^24^.

The reduced effort exertion for low-incentive level in high anxiety seems to be at odds with the broad literature from the cognitive domain indicating that high-anxious individuals tend to apply “compensatory strategies” (e.g., increases in effort or in use of neural resources) to boost performance (for reviews, see refs. ^14,18,76^). This suggests that in healthy individuals, a high-anxious trait might be able to develop ad hoc adaptations, requiring the exertion of either more (e.g., for demanding cognitive tasks) or less (e.g., for demanding physical tasks) effort to keep up with performance at equivalent levels as low-anxious ones. That less effort exertion might be a “compensatory strategy” is suggested by the increased allocation of neural activity (in terms of CNV amplitude and ACC activity) in high-anxious participants for this particular, low incentive condition.

Our EEG results provide unique insights into the temporal brain dynamics related to the processing of the different incentive levels, identifying two distinct time windows in the temporal sequence of reward anticipation. First, in line with previous findings^39,41,77^, the CNV amplitude for the interval ranging from −2115 to −1345 ms to EMG onset was sensitive to monetary incentive levels. In agreement with previous reports^40,41,77^, we found that higher incentives elicited a larger CNV than the low incentive condition. Our subsequent source analyses indicated increased activity in the ACC for the high-incentive level, while in the PCC for the low incentive level. Enhanced PCC activity is consistently observed in resting state conditions and in nonengaging tasks^78^, suggesting that its involvement in our task is possibly due to reduced mental and preparatory efforts. The identification of increased ACC activity triggered by the highest incentive is in line with the former implication of this brain region in anticipatory reward processing^79^. Indeed, ACC neurons are known to respond to reward^80^, and to discriminate rewarding from nonreward-predicting stimuli^81^. Accordingly, the ACC can help guiding behavior by integrating information regarding individual states like motivation and reward encoding with motor preparation and response execution^79^.

Importantly, we found a significant interaction between trait anxiety and incentive level on CNV modulation at the intervals ranging from −2610 to −2510 ms. This finding is consistent with the view that anxiety is associated with higher CNV amplitudes across various cognitive tasks^17,44,46^. Remarkably, we found greater CNV responses to the lower monetary incentive sizes in high- anxiety, and being the condition where we find the key grip force differences between the high- and low-anxiety groups. This finding is consistent with the evidence that anxious individuals show augmented CNV amplitudes during the anticipation of negative information^44^.

Interestingly, our mediation analysis identified the ACC as the only significant mediator in the relationship between trait anxiety and ΔGrip. The increased involvement of the ACC in the ‘economy’ of effort observed in high-anxious individuals supports the view that their behavior corresponds to a compensatory strategy (see the “limited resources” discussion above). It also aligns with several of the reported roles for this brain region. Thus, in addition to strong evidence implicating the ACC in effort evaluation^35,48,82^, ACC neurons are believed to encode information about rewards, effort costs, and actions^83–88^, integrating information into an economic value signal (see ref. ^49^). In addition, microstimulating the dorsal ACC has been shown to evoke a subjective sense of preparation to overcome a challenge^89^. Recently, a neural signature of decision-making for choices involving motor costs was identified in the human cingulate cortex more broadly^49^. Furthermore, the ACC has also been implicated in regulating the conflict between goals and distractors^90,91^, which in high-anxious individuals would typically involve worry or self-preoccupation regarding (among others) failure in evaluative or competitive situations^18^. In this regard, it is important to note that the effect was lateralized to the right hemisphere, and that the right ACC is believed to play a critical role in modulating adaptive behavioral responses^92,93^. Right ACC structural abnormalities have been found to be associated with impulsive/aggressive behaviors during development^94,95^, especially in boys^94^. Therefore, our findings provide additional insights into the relationship between the ACC and trait anxiety.

This study has several limitations. It was carried out in 19–30-year-old male participants only with a modest sample size, limiting the generalization of the findings to the general population. In addition, our study was carried out in healthy individuals and any potential clinical implication remains to be established.

To conclude, we present strong evidence for trait anxiety-related differences in incentive processing in an effortful monetary-incentivized task. Few studies have already reported alterations in neural responses to monetary incentives in several psychiatric disorders, such as addiction, schizophrenia and depression^30^. Indeed, an abnormal increase in ACC activation has been found in unmediated depressed patients during monetary incentive processing in anticipation of gains^96^. However, only very few studies have specifically investigated the impact of anxiety disorders on reward anticipation (e.g., refs. ^97,98^). Given that high-trait anxiety constitutes a risk factor for the development of depression^2,3^, it would be important that future studies investigate whether our reported enhanced ACC engagement between trait anxiety and the differential “energization” of behavioral responses in a monetarily incentivized motor task might be a biomarker for individuals at risk for depression.

## Supplementary information

supplemental material
