# Supplementary material for: Trait anxiety on effort allocation to monetary incentives: a behavioral and high-density EEG study
Source: Transl Psychiatry. 2019 Jul 12;9:174. doi: 10.1038/s41398-019-0508-4 (PMC6626005; doi:10.1038/s41398-019-0508-4)
Supplement: Supplementary file 1 — supplemental material [file 41398_2019_508_MOESM1_ESM.pdf]

Supplementary Material for

Trait anxiety on effort allocation to monetary incentives: A behavioral and high-density EEG study

Cristina Berchio*, João Rodrigues*, Alina Strasser, Christoph M. Michel, Carmen Sandi

*These authors contributed equally to this work.

This file includes:

Supplementary Methods:

- Pilot study and power analysis
- Mediation analysis

Supplementary results:

- State anxiety effects on MID parameters

Table S1

Figure S1

References

Supplementary Methods

Participants

Sixteen healthy adult individuals were recruited to the study. This number was determined with a power analysis with data from a previous pilot experiment to achieve a power of 0.95 and Type I error rate of 0.05 (see Power analyses section below). Recruitment criteria were the following: male, 19-30 years old, right-handed, non-smoker, no psychiatric or neurological illness, no sleep deprivation the day before the recording, no history of brain or skull surgery, good visual acuity or corrected for vision. Table 1 contains participants' phenotypic characteristics. Experiments were performed between noon and 6 pm. Participants were instructed not to eat or to drink any caffeinated drinks one hour before the experiment. Furthermore, participants were instructed not to take any medication and to avoid physical effort within 24 hours before the experiment. The study was approved by the Cantonal Ethics Committee of Geneva, Switzerland. Informed written consent was obtained from all participants.

Psychometric questionnaires

State and trait anxiety were assessed with the State-Trait Anxiety Inventory (STAI) [1]. Participants' fatigue (state and trait) was assessed with the Mental and Physical State Energy and Fatigue Scales (SEF) [see [2, 3]]. The trait-anxiety score was used to median-split the data into high and low trait-anxiety groups. The experimenter was blind to the experimental group of each participant.

Modified Monetary Incentive Delay task

The behavioral task used relies on exerting force on a hand dynamometer (TSD121B-MRI, Biopac) at a particular threshold from the maximum voluntary contraction (MVC). To set individuals' task threshold, each participant was instructed to exert as much force as possible on the dynamometer for 1 sec, repeated 3 times interspersed by 3 min breaks to allow for recovery before each MVC measurement. The highest MVC out of the three values exerted by each participant was used to calibrate the threshold for the handgrip force required to be exerted for that participant in the behavioural task. The signal recorded with the dynamometer, linearly proportional to the exerted force (in kg), was fed back to the stimuli presentation PC (running the MID task in E-Prime) in real-time.

Participants performed a modified version of the monetary incentive delay (MID) task [4]. Monetary incentivized trials started with a fixation cross (0.6 sec), followed by an anticipatory period (3.5 sec) indicating the potential gain: winning CHF 1, CHF 0.5 or CHF 0.2 (90 total incentivized trials). To earn these monetary incentives, participants were instructed to exert force on a hand dynamometer. The beginning of the force exertion period was signalled by the appearance of a red circle around the fixation cross. If a certain threshold [i.e., 40% of the participant's MVC force] was reached within 2 sec the red circle was replaced by a green circle. The green circle also indicated that participants had to maintain the contraction force level above a maintenance threshold (40% of MVC force -0.5 kg) for another 3 sec. If participants did not reach the threshold in the initial 2 sec or if the force level fell below the maintenance threshold during the 3 sec endurance period, the trial was failed, visualized by a red

cross occurring on the screen for 1 sec. Trial success was indicated by a green tick (1 sec) in the absence of the central fixation cross (see Figure 1 in main text). To avoid preparatory biases during the fixation period, 30 resting trials, without monetary incentives, were also included. For these trials, subjects were instructed that when a blurred (coin) stimulus appeared they could rest. The experiment was split into three blocks of 8 min each, separated by a 3 min inter-block break. The presentation order of monetary incentives varied pseudo-randomly within each block. Thus, 120 trials were presented in a pseudo-random order. Before performing the actual experiment, to ensure participants understood the task, they completed a 20 trial training session. Last, participants were inquired for their perceived force threshold used during the trials to turn the circle green, on a scale from 10% to 120% of their MVC with steps of 10%.

During task performance, EEG recordings were obtained from each participant while seating in a comfortable position and in front of a computer screen at 90 cm distance. The participant's right forearm rested on an arm mount (fixed to the stimulus presentation table) to reduce movement artefacts.

Behavioural measures

Accuracy was calculated for each incentive level as the number of successful responses (i.e., achieving the 40 % MVC threshold within 2 seconds and keeping above that value for an additional 3 sec) divided by the number of total trials for each incentive level. Subject's raw grip force was normalized by their MVC (in kg) and total grip force calculated by the area under the curve (AUC) during the maximum allowed response period (2 sec).

Response latencies were calculated for trials in which the 40% MVC threshold was reached and it represents the latency after the imperative signal until the MVC reached 5%. Variables representing the difference in behavior between the highest (1 CHF) and lowest (0.2 CHF) incentive level were calculated for accuracy, grip force and response latency and respectively termed ΔAccuracy, ΔGrip, and ΔLatency.

Further, because stress has been shown to increase confidence in low anxious individuals while decreasing it in high anxious [5], behavioral self-confidence was measured on a 7-point rating scale before each block, and to be used as a confounding variable. This provided an index of participants' confidence to win the maximum amount possible during the task. Salivary cortisol was also measured (see methods below).

EEG recordings and pre-processing

Scalp EEG was continuously recorded from 256 electrodes at 1000 Hz sampling rate (Electrical Geodesics Inc., Oregon). Data were acquired with a band pass filter of 0.01 - 1000 Hz, and electrodes impedances were kept below 30 k-ohms. EEG data and electromyogram (EMG) activity were simultaneously acquired (Physio16 input box, Geodesic EGI Inc.). EMG activity was recorded from the flexor muscles of the right harm.

In order to identify the CNV, data were averaged across trials aligned to movement onset determined by the EMG. For that, the EMG signals were filtered offline (20 - 50 Hz, Butterworth filter), and rectified with a time constant of 500 ms. EMG onset was defined by the instant at which the amplitude response deviated greater than 3 standard deviations away from the background period.

EEG pre-processing was performed using Cartool Software (https://sites.google.com/site/cartoolcommunity/) [6]. Off-line, data were reduced to 204 channels [see [7]], band-pass filtered between 0.1 to 1 Hz (second order causal Butterworth filter, 24db/octave roll-off), and down-sampled to 250 Hz. We selected this filter setting based on a previous study that showed that a band-pass filter of 0.1-1 Hz best separates slow cortical potentials between go- and no-go trials [8] as well as movement intention [9] on a single-trial level. After downsampling and filtering, artifactual electrodes were visually detected and interpolated using a 3D spherical spline [10] (Perrin et al., 1987). Relative to the EMG onset, EEG epochs were segmented from −3000 to + 400 ms. Trials exceeding ±100υV and contaminated by artefacts were rejected. Individual data were then re-referenced to an average reference. In total, five percent of the trials had to be rejected; the number of rejected trials did not differ between incentive conditions (two-way ANOVA, with 'incentive' as repeated measures, main effect: $F_{1,15} = 1.37$; $p = 0.269$).

Hormonal responses

Participants' saliva was collected to analyze salivary cortisol levels at both baseline and changes taking place during the experiment. Samples were taken four times: upon arrival at the laboratory, before the behavioral task, after the behavioral task and once all experimental procedures were completed. Participants were instructed to place a cotton swab (SCS Salimetrics) medially under the tongue for 2 min. Samples were immediately stored on dry ice and then frozen at -20°C until processed. Samples were then centrifuged at 3000 rpm for 15 min at room temperature, and salivary cortisol concentrations were measured by enzyme immunoassay according to the manufacturer's instructions (Salimetrics, Newmarket, Suffolk, United Kingdom). To estimate overall cortisol concentration and reactivity across the experiment, we computed the area under the curve with respect to ground (AUCg) and with respect to increase (AUCi) indices [11]. Cortisol AUCi represents overall changes in cortisol concentration (i.e., how sensitive the stress system is to the experimental manipulation) while cortisol AUCg represents total hormonal output throughout the experiment. Saliva samples were not measured for one subject.

Statistics: Behavior and Cortisol

For behavior (accuracy, grip force and response latency), repeated measures ANOVA (rm-ANOVA) was used to test for the effect of incentives (CHF 0.2, 0.5 and 1 as within-subjects variables). Mixed-design ANOVAs were used to test for the interaction between incentives (as within-subjects variable) and trait-anxiety (trait-anxiety group as between-subjects variable) on behavior. To assess the interaction between incentive and state-anxiety on behavior, repeated measures ANCOVAs (rm-ANCOVAs) with state-anxiety scores as a covariate were used. For hormonal variables, MVC or other variables not belonging to an incentive level, ANOVA was used to test the main effect of trait-anxiety group and Spearman correlations were used to test the effect of state-anxiety.

Following a significant effect of incentives when using rm-ANOVA, *post hoc* tests were employed using paired *t*-tests on the different levels of the incentive variable. If an interaction between trait-anxiety group and incentives was significant, simple main effects of incentives were tested for each anxiety group. Significant simple main effects of incentives were followed by *post hoc* tests employing paired *t*-tests on the different levels of the incentive variable. When the interaction between state-anxiety and incentives was significant, *post hoc* Spearman correlations were applied between the state-anxiety score and the behavioral variable, for each level of incentives. Due to the limited sample size, all reported correlations follow the Spearman rank ρ statistic in order to better control for Type I errors; full details (normality test, ρ coefficient, *p*-value, confidence intervals and scatterplot) can be consulted in Table S1.

Holm correction was applied to *p*-values from multiple comparisons in *post hoc* tests and simple effects while false discovery rate (FDR) correction was applied for planned comparisons. Sphericity assumption violation was assessed with Maulchly's test of sphericity and whenever necessary, Greenhouse-Geisser correction was applied to the *F*-distribution's degrees of freedom. All statistical tests for behavior and cortisol variables were performed in JASP version 0.9 (https://jasp-stats.org) and R for multiple comparison corrections.

Electrical neuro-imaging analyses

The CNV was analyzed using spatio-temporal methods [12-14]. To assess the strength of the responses to incentive levels at each time point, we used the global field power (GFP). The GFP measures the global strength of the electric field as the standard deviation of all potentials referred to the average reference [10, 15, 16]. It can be considered as a global measure of neuronal synchronization [17]. Differences of the GFP between the three incentive conditions were evaluated time-point by time-point using the RAGU software [18] with an analysis of covariance (ANCOVA) with incentives (CHF 0.2, CHF 0.5 or CHF 1) as main effect and anxiety as the covariate (trait-anxiety/state-anxiety scores). To prevent false positive rates, due to multiple comparisons across time, permutation tests were performed [19]. In addition a *post hoc* analysis was applied to the average GFP of the identified time windows of significance [see [18]].

Source localization of the CNV was performed with Cartool Software [6] using a distributed linear inverse solution ([LAURA, [20]]. The forward model employed uses 5018 voxels within the grey matter of an average brain template [21]. The lead field was calculated using the LSMAC head model (Brunet et al., 2011). After the inverse matrix was applied, for each participant, each voxel was standardized individually with its on baseline (i.e., mode activity across the whole time range).

Differences in brain activity were investigated for the average time windows where the scalp data analysis was significant. For these windows, contrast analyses between the incentives were performed using randomization tests (5000 permutation runs, *p* values of 0.05 or less) [see [19]] in 80 critical region-of-interest (ROI) [AAL-based parcellation [22]]. Unpaired t tests (two-tailed, $p \leq 0.05$) were applied for significant ROIs in order to determine the direction of the significant effect.

When covariant effects were significant, explorative *post-hoc* analyses were performed (see section Moderation and Mediation) using the ROI identified by contrast analysis of incentives. These ROI's

maximal current densities, extracted at the temporal windows where trait/state anxiety were significant covariant, were entered into models predicting behavior from trait-anxiety group, both as moderators and mediators.

Moderation and Mediation analyses

To gain insight into whether and how brain activations affect the relationship between anxiety and behavior, moderation and mediation models were used.

The moderation analyses included a regression model containing i) the behavior of interest as the response variable, ii) the moderation between the brain activation of interest and trait-anxiety, and iii) other relevant confounding variables. For our behavioral parameters, model testing was done on each identified ROI (i.e., main contrast between monetary incentives). The Bonferroni correction procedure was applied to the *p*-value of each regressor, correcting for the number of models tested for the same response variable.

Similarly, we performed mediation analyses to test if any of the identified ROIs mediates the relation between trait-anxiety and behavior. Elucidating this effect required the identification of a causal chain of trait-anxiety predicting the CNV-related ROI activations, which in turn predict behavior. To test our mediation hypotheses, we used the R package 'Mediation' [23]. This package uses a quasi-Bayesian Monte Carlo approximation method in which the posterior distribution of quantities of interest is approximated by their sampling distribution [24]. As it is recommended to use a large number of simulations for small data sets, we used 10000 simulations for each model. This analysis uses two regression models that were fitted separately: the mediator and the outcome model. In the mediator model, the mediator (the CNV activation at one ROI) is predicted by the treatment variable (trait-anxiety group) and a set of observed pretreatment confounders. In the outcome model, the outcome variable is predicted by the mediator, the treatment variable and the set of observed pretreatment confounders.

These pretreatment confounders are important for the assumption of sequential ignorability (under which, the average causal mediation effects are non-parametrically identified). Briefly, sequential ignorability consists of two assumptions [24]: i) the treatment variable is independent of all potential values of the outcome and mediating variables (conditional on the pretreatment confounders); and ii) the mediator is independent of all potential outcomes given the observed treatment and pretreatment confounders. Linear model assumptions, skewness, kurtosis, and heteroscedasticity were checked for the mediator and output regression models with the R package 'gvlma' [25]. A heteroscedasticity-consistent estimator for the covariance matrix, with small sample bias adjustment, from the 'sandwich' package (HC3 type in option 'vcovHC') [26] was used for the mediation analyses.

We checked for the following mediation indicators: average causal mediation effects (ACME), average direct effects (ADE), combined indirect and direct effects (Total Effect) and the ratio of ACME by ADE (Prop. Mediated). Significant ACME values indicate a significant mediation effect. As with the moderation analysis, we tested each ROI activation as a mediator on a dedicated model, for each behavior of interest and corrected the p-values of the ACME and ADE values using the Bonferroni correction procedure, correcting for the number of tested models.

Since sequential ignorability is a strong assumption (i.e., it excludes the existence of -measured or unmeasured- posttreatment covariates, as well as that of unmeasured pretreatment covariates), a sensitivity analysis is recommended to assess the possible existence of unobserved pretreatment covariates [23]. The correlation ρ between the residuals of the mediator and outcome regression models was chosen as the sensitivity parameter. This value informs about how large departures from the ignorability assumption of the mediator can be, until the ACME is not reliable (i.e. how robust our mediation effect is to unmeasured confounders).

Pilot study and power analysis

In order to calculate the necessary sample size for a power of 95% in our study, we analyzed data from a pilot study performed at the Ecole Polytechnique Federale de Lausanne by the same authors. The pilot study involved 33 participants that performed the modified MID task (see Methods in main text) that were split into low and high anxiety groups according to the median value (median STAI-T score = 37) (low trait-anxiety group: N=15, *M*=31.7 ± 4.11; high trait-anxiety group: N=11, *M*=45.64 ± 6.52). The task included three sequential challenge conditions (i.e., playing on his own, playing against another participant, or playing against 15 other participants). Data from 7 participants had to be excluded due to missing values, resulting in a final total of 26 participants.

We considered that the arousing nature for participants of experiments involving high-density EEG recordings would be comparable to the arousal elicited by the intermediate challenging condition in our pilot involving a competition against another participant. First, in the pilot data, we tested a model predicting grip force with one between-subjects variable (trait-anxiety group) and two within-subjects factors: 'incentive' (CHF 0.2, CHF 0.5 and CHF 1) and 'competitive challenge' ("alone", "versus one", "versus 15") with a mixed design ANOVA. Given the triple interaction term in this ANOVA ($F_{4, 96} = 2.42$, $p = 0.054$, $\eta^2 = 0.095$), we tested models for each challenge using a mixed design ANOVA with trait-anxiety group as between-subjects factor and incentive as within-subjects factor (correction of *p*-values was not employed). The interaction term anxiety-group x incentive was significant ($F_{2, 48} = 3.28$, $p = 0.046$, $\eta^2 = 0.091$) for the "versus one" challenge (*p*s > 0.243 for remaining challenges). Simple main effects revealed that there was a significant effect of incentive on grip force only for the high trait-anxiety group ($p = 0.002$) where high higher rewards lead to stronger grip force. The low trait-anxiety group showed no effects of incentive on grip force ($p = 0.429$). The null-hypothesis of equal variances was not rejected using the Levene's test of equal variances both for the between and within-subjects variables (all *p*s ≥ 0.132).

Then, we used the G*Power v3.1.9.2 software to calculate the necessary total sample size to observe the group x incentive interaction effect on grip force with a Type I error rate of 0.05 (α=0.05) and a power of 0.95 (1-β=0.95). The required statistical test is referred in the G*Power options as "ANOVA: Repeated measures, within-between interaction". The type of power analysis chosen was "A priori: Compute required sample size – given alpha, power and effect size". The calculation of the "Effect size f" is required from our experiment's interaction partial η^2. This value was calculated in JASP (partial $\eta^2 = 0.120$). This is an average to high effect size if we follow the conventions for partial η^2 values: small = 0.02; medium = 0.06; large = 0.14. The "Effect size f" was then calculated in G*Power (f = 0.369) and

inputted in the main power analysis. Another required choice for the analysis is the expected correlation among repeated measures in the "versus one" challenge. Here we observed values larger than 0.9 (min r = 0.953) however, we inputted 0.8 for this analysis, as a safety margin. Finally, we chose the "Nonsphericity correction ε" value. This value is 1.0 if sphericity assumption is expected to be met or lower if otherwise, with the lowest value being 0.5 in our case (lowest value = 1/[repetitions – 1]). With these settings, the estimated necessary total sample size was defined as 16 and a critical F score of 4.60.

Specifically, the input and resulting output from the G*Power software for this analysis was:

F tests ANOVA: Repeated measures, within-between interaction
Analysis A priori: Compute required sample size

Input Effect size f = 0.3692745
α err prob = 0.05
Power (1-β err prob) = 0.95
Number of groups = 2
Number of measurements = 3
Corr among rep measures = 0.8
Nonsphericity correction ε = 0.5
Output Noncentrality parameter λ = 16.365
Critical F = 4.6001099
Numerator df = 1.00
Denominator df = 14.00
Total sample size = 16
Actual power = 0.9636689

Supplementary Results

State-anxiety and Accuracy

Although there was no significant main effect of state-anxiety on accuracy (Figure S1a, $F_{1, 14} = 2.44$, $p = 0.141$, $\eta^2 = 0.148$), state-anxiety showed a significant interaction with incentive (Figure S1a, $F_{2, 28} = 16.77$, $p < 0.001$, $\eta^2 = 0.425$) but a non-significant positive correlation with ΔAccuracy [see Figure S1b ρ(14) = 0.44, $p = 0.087$]. No significant correlations between accuracy in different incentive levels and state-anxiety were found (all *p*s > 0.375; see Table S1).

State-anxiety and Grip force

In order to explore the specificity of trait-anxiety on the identified effects, we also assessed the potential relationship between state-anxiety and grip force. However, there was no significant main effect (see Figure S1c; $F_{1, 14} = 0.53$, $p = 0.480$, $\eta^2 = 0.036$), nor an interaction between state-anxiety and incentives (see Figure S1c; $F_{1.25, 17.46} = 2.13$, $p = 0.160$, $\eta^2 = 0.130$). There was also no significant association between state-anxiety scores and ΔGrip [see Figure S1d; ρ(14) = 0.43, $p = 0.092$].

State-anxiety and response latency

No interaction between incentives and state-anxiety (see Figure S1e; $F_{1.37, 19.21}$ = 0.1.15, $p = 0.317$, $\eta^2 = 0.070$) were found for response latencies, nor significant main effects of state-anxiety (see Figure S1e, a;$F_{1, 14} = 0.01$, $p = 0.917$, $\eta^2 < 0.001$). State-anxiety was also not associated with ΔLatency (see Figure S1f; ρ(14) = 0.06, $p = 0.827$).

Table S1: Supplementary robust correlations. Details from all correlations performed in this study. In order to have better control over Type I error probability, we use Spearman rank ρ correlations [27]. Winsorized Pearson correlations are also presented on the table for comparison, since the Spearman correlation is a rank based measure. Data were winsorized at 20% before computing the Pearson correlation coefficient, as recommended in [27] for small sample sizes and to avoid possible loss of power of other robust correlation methods like the skipped correlation [27]. The Matlab toolbox *robustcorrtool*[28] was used to compute correlation coefficients, *p*-values and bootstrapped 95% confidence intervals (CI) and to test for multivariate normality. Winsorization of data was performed with in house code. *[a] the Henze-Zirkler multivariate normality test was used to test the null hypothesis that the variable pairs are normal. *[b] FDR correction is used for all *p*-values except when the correlation tests result from a post-hoc test (3 corrlations between state-anxiety and accuracy at each of the 3 reawrd levels), when a Holm correction is used. Correction was applied to each group of variables within the same contiguous non-delimieted rows.

**Variable x**	**Variable y**	***N***	***Multivariate Normality Test *[a]***	***Spearman (ρ), Pearson (r) Correlation***	***p*-value (two-tailed)**	***p*-value corrected (FDR*[b])**	***Bootstrapped 95% CI***	
**Trait-anxiety**	**Trait physical fatigue**	16	0.305	ρ=0.62	0.011	0.011	[0.12 0.86]	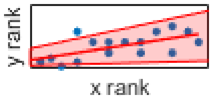
				r=0.59	0.016	0.016	[0.16 0.86]	
**Trait-anxiety**	**Trait mental fatigue**	16	0.305	ρ =0.81	<0.001	<0.001	[0.50 0.95]	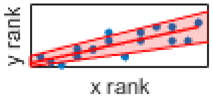
				r=0.74	0.001	0.002	[0.51 0.90]	
**Self-confidence**	**Grip force CHF 0.2**	16	0.825	ρ =0.58	0.018	0.036	[0.09 0.87]	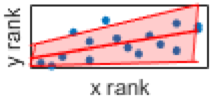
				r=0.56	0.023	0.046	[0.11 0.87]	
**Self-confidence**	**Grip force CHF 1**	16	0.825	ρ =0.17	0.523	0.523	[-0.42 0.68]	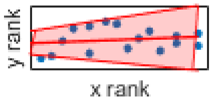
				r=0.23	0.394	0.394	[-0.43 0.65]	
**Trait-anxiety**	**ΔAccuracy**	16	0.305	ρ =0.12	0.658		[-0.43 0.64]	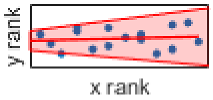
				r=0.18	0.498		[-0.44 0.63]	

**State-anxiety**	**Accuracy CHF 0.2**	16	0.011	ρ =-0.40	0.125	0.375	[-0.84 0.19]	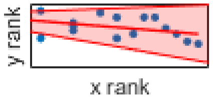
				r=-0.52	0.039	0.117	[-0.91 0.27]	
**State-anxiety**	**Accuracy CHF 0.5**	16	0.011	ρ =0.17	0.527	0.834	[-0.35 0.74]	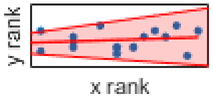
				r=0.01	0.971	1.000	[-0.63 0.66]	
**State-anxiety**	**Accuracy CHF 1**	16	0.011	ρ =0.22	0.417	0.834	[-0.32 0.68]	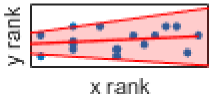
				r=0.13	0.637	1.000	[-0.44 0.62]	
**State-anxiety**	**ΔAccuracy**	16	0.011	ρ =0.44	0.087		[-0.17 0.85]	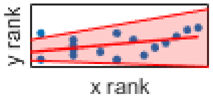
				r=0.62	0.011		[0.02 0.89]	
**ΔGrip**	**Trait-anxiety**	16	0.177	ρ =0.75	0.001		[0.48 0.91]	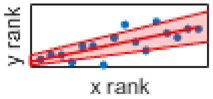
				r=0.74	0.001		[0.51 0.92]	
**ΔGrip**	**State-anxiety**	16	0.177	ρ =0.43	0.092		[-0.15 0.83]	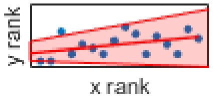
				r=0.37	0.164		[-0.15 0.73]	
**ΔGrip**	**ΔAccuracy**	16	0.177	ρ =-0.13	0.636		[-0.70 0.42]	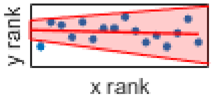
				r=-0.09	0.732		[-0.68 0.42]	
**Grip force CHF 0.2**	**Accuracy CHF 0.2**	16	0.253	ρ =0.17	0.533	0.535	[-0.41 0.66]	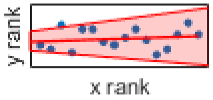
				r=0.10	0.725	0.823	[-0.45 0.59]	
**Grip force CHF 0.5**	**Accuracy CHF 0.5**	16	0.895	ρ =-0.17	0.527	0.535	[-0.66 0.40]	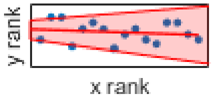
				r=-0.06	0.823	0.823	[-0.62 0.38]	

**Grip force CHF 1**	**Accuracy CHF 1**	16	0.473	ρ =-0.17	0.535	0.535	[-0.69 0.44]	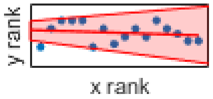
				r=-0.14	0.610	0.823	[-0.69 0.39]	
**ΔLatency**	**Trait-anxiety**	16	0.608	ρ =0.11	0.695		[-0.43 0.59]	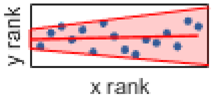
				r=0.09	0.750		[-0.47 0.48]	
**ΔLatency**	**State-anxiety**	16	0.608	ρ =-0.06	0.827		[-0.63 0.50]	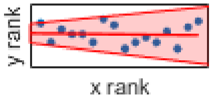
				r=0.01	0.967		[-0.56 0.45]	
**ΔLatency**	**ΔAccuracy**	16	0.608	ρ =0.15	0.586	0.966	[-0.37 0.61]	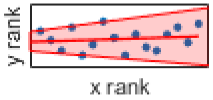
				r=0.13	0.624	0.990	[-0.35 0.57]	
**ΔLatency**	**ΔGrip**	16	0.608	ρ =0.01	0.966	0.966	[-0.55 0.63]	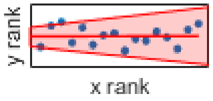
				r=0.00	0.990	0.990	[-0.54 0.58]	

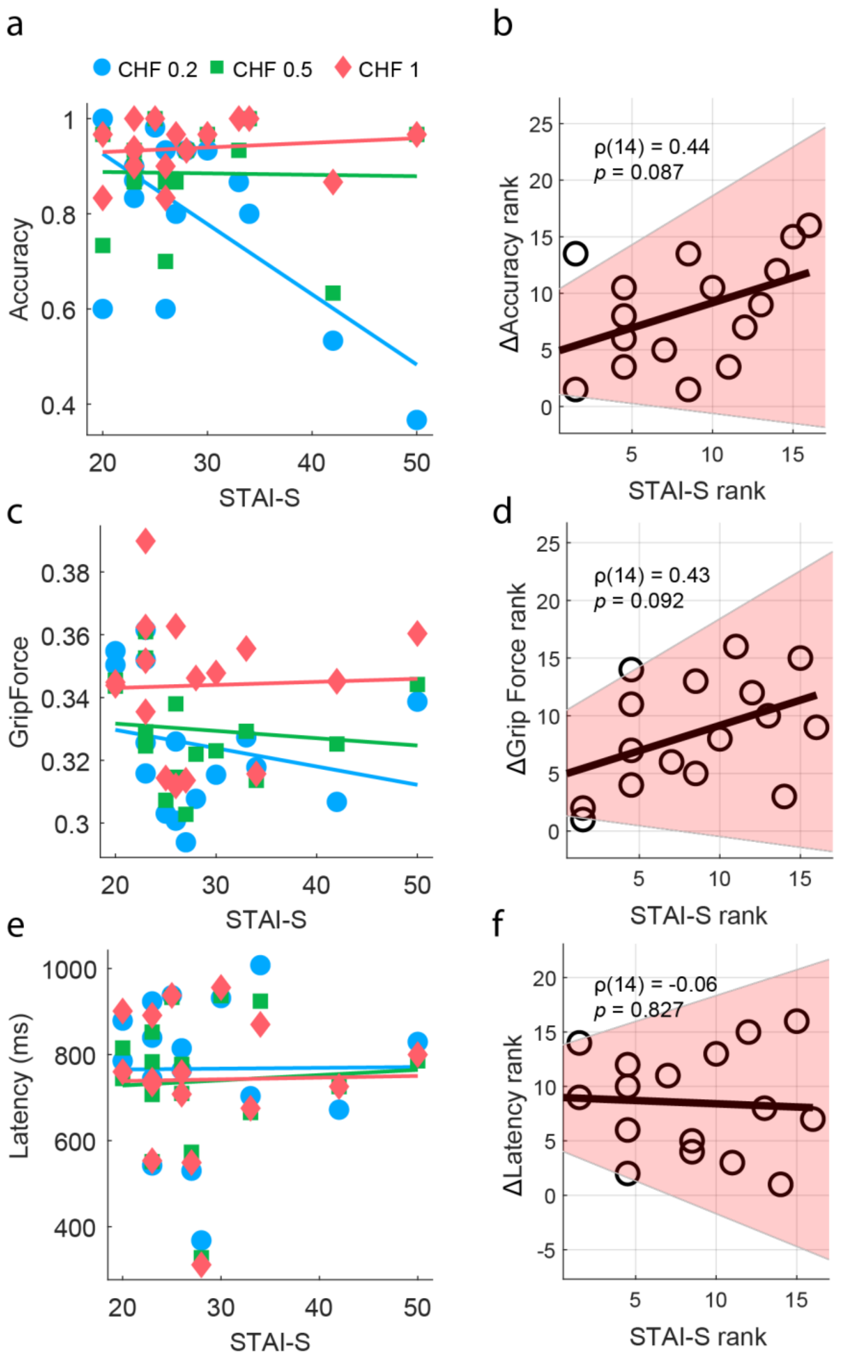

Figure S1: Behavioral effects of incentive level and state-anxiety: a) No significant main effect of trait-anxiety but a significant interaction effect between state-anxiety and incentive level were observed. b) Correlation between the variable ΔPerformance rank and state-anxiety scores. 95% CI within shaded area. c) No significant main effect of trait-anxiety nor significant interaction effect between state-anxiety and incentive level d) Correlation between the variable ΔGrip and state-anxiety scores. 95% CI within shaded area. e) No significant interaction or main effects were present for state-anxiety on latency. i) Correlation between the variable ΔLatency and state-anxiety scores. 95% CI within shaded area.

References

1. Spielberger CD. *State-trait anxiety inventory: a comprehensive bibliography*. Consulting Psychologists Press, 1989.

2. Loy BD, O'Connor PJ. The effect of histamine on changes in mental energy and fatigue after a single bout of exercise. *Physiology & behavior* 2016; **153:** 7-18.

3. O'Connor P. Mental and physical state and trait energy and fatigue scales. *University of Georgia, Athens, GA, USA Personal communication* 2004.

4. Knutson B, Adams CM, Fong GW, Hommer D. Anticipation of increasing monetary reward selectively recruits nucleus accumbens. *Journal of Neuroscience* 2001; **21**(16)**:** RC159-RC159.

5. Goette L, Bendahan S, Thoresen J, Hollis F, Sandi C. Stress pulls us apart: anxiety leads to differences in competitive confidence under stress. *Psychoneuroendocrinology* 2015; **54:** 115-123.

6. Brunet D, Murray MM, Michel CM. Spatiotemporal analysis of multichannel EEG: CARTOOL. *Computational intelligence and neuroscience* 2011; **2011:** 813870.

7. Berchio C, Rihs TA, Piguet C, Dayer AG, Aubry JM, Michel CM. Early averted gaze processing in the right Fusiform Gyrus: An EEG source imaging study. *Biological psychology* 2016; **119:** 156-170.

8. Garipelli G, Chavarriaga R, Millan Jdel R. Single trial analysis of slow cortical potentials: a study on anticipation related potentials. *Journal of neural engineering* 2013; **10**(3)**:** 036014.

9. Lew E, Chavarriaga R, Silvoni S, Millan Jdel R. Detection of self-paced reaching movement intention from EEG signals. *Frontiers in neuroengineering* 2012; **5:** 13.

10. Perrin F, Pernier J, Bertrand O, Giard MH, Echallier JF. Mapping of scalp potentials by surface spline interpolation. *Electroencephalography and clinical neurophysiology* 1987; **66**(1)**:** 75-81.

11. Pruessner JC, Kirschbaum C, Meinlschmid G, Hellhammer DH. Two formulas for computation of the area under the curve represent measures of total hormone concentration versus time-dependent change. *Psychoneuroendocrinology* 2003; **28**(7)**:** 916-931.

12. Michel CM, Murray MM. Towards the utilization of EEG as a brain imaging tool. *NeuroImage* 2012; **61**(2)**:** 371-385.

13. Michel CM, Murray MM, Lantz G, Gonzalez S, Spinelli L, de Peralta RG. EEG source imaging. *Clinical neurophysiology* 2004; **115**(10)**:** 2195-2222.

14. Murray MM, Brunet D, Michel CM. Topographic ERP analyses: a step-by-step tutorial review. *Brain topography* 2008; **20**(4)**:** 249-264.

15. Lehmann D, Skrandies W. Reference-free identification of components of checkerboard-evoked multichannel potential fields. *Electroencephalography and clinical neurophysiology* 1980; **48**(6)**:** 609-621.

16. Michel CM, Brandeis D, Skrandies W, Pascual R, Strik WK, Dierks T*, et al*. Global Field Power: a 'time-honoured' index for EEG/EP map analysis. *International journal of psychophysiology : official journal of the International Organization of Psychophysiology* 1993; **15**(1)**:** 1-5.

17. Skrandies W. Global field power and topographic similarity. *Brain topography* 1990; **3**(1)**:** 137-141.

18. Koenig T, Kottlow M, Stein M, Melie-García L. Ragu: a free tool for the analysis of EEG and MEG event-related scalp field data using global randomization statistics. *Computational intelligence and neuroscience* 2011; **2011:** 4.

19. Maris E, Oostenveld R. Nonparametric statistical testing of EEG-and MEG-data. *Journal of neuroscience methods* 2007; **164**(1)**:** 177-190.

20. de Peralta Menendez RG, Andino SG, Lantz G, Michel CM, Landis T. Noninvasive localization of electromagnetic epileptic activity. I. Method descriptions and simulations. *Brain topography* 2001; **14**(2)**:** 131-137.

21. Collins DL, Neelin P, Peters TM, Evans AC. Automatic 3D intersubject registration of MR volumetric data in standardized Talairach space. *Journal of computer assisted tomography* 1994; **18**(2)**:** 192-205.

22. Tzourio-Mazoyer N, Landeau B, Papathanassiou D, Crivello F, Etard O, Delcroix N*, et al*. Automated anatomical labeling of activations in SPM using a macroscopic anatomical parcellation of the MNI MRI single-subject brain. *NeuroImage* 2002; **15**(1)**:** 273-289.

23. Tingley D, Yamamoto T, Hirose K, Keele L, Imai K. Mediation: R package for causal mediation analysis. *Journal of Statistical Software* 2014; **59**(5).

24. Imai K, Keele L, Tingley D. A general approach to causal mediation analysis. *Psychological methods* 2010; **15**(4)**:** 309-334.

25. Pena EA, Slate EH. Global Validation of Linear Model Assumptions. *Journal of the American Statistical Association* 2006; **101**(473)**:** 341.

26. Zeileis A. Object-oriented computation of sandwich estimators. *Journal of Statistical Software* 2006; **16:** 1-16.

27. Wilcox RR. Comparing dependent robust correlations. *The British journal of mathematical and statistical psychology* 2016; **69**(3)**:** 215-224.

28. Pernet CR, Wilcox R, Rousselet GA. Robust correlation analyses: false positive and power validation using a new open source matlab toolbox. *Frontiers in psychology* 2012; **3:** 606.
